# Understanding PrEP misconceptions and their impact on PrEP initiation and use among pregnant and lactating women in Malawi

**DOI:** 10.1371/journal.pgph.0004667

**Published:** 2025-05-27

**Authors:** Alinda M. Nyamaizi, Marley Catlett, Friday Saidi, Twambilile Phanga, Jennifer Tseka, Agatha Bula, Lisa D. Pearce, Suzanne Maman, Carol E. Golin, Wilbroad Mutale, Benjamin H. Chi, Lauren M. Hill

**Affiliations:** 1 Department of Maternal and Child Health, University of North Carolina at Chapel Hill, Chapel Hill, North Carolina, United States of America; 2 RTI International, Research Triangle Park, Chapel Hill, North Carolina, United States of America; 3 Department of Health Behavior, University of North Carolina at Chapel Hill, Chapel Hill, North Carolina, United States of America; 4 UNC Project-Malawi, Lilongwe, Malawi; 5 Department of Obstetrics and Gynecology, School of Medicine, University of North Carolina at Chapel Hill, Chapel Hill, North Carolina, United States of America; 6 Department of Sociology, University of North Carolina at Chapel Hill, Chapel Hill, North Carolina, United States of America; 7 Department of Medicine, University of North Carolina at Chapel Hill, Chapel Hill, North Carolina, United States of America; 8 Department of Health Policy, University of Zambia School of Public Health, Lusaka, Zambia; Simon Fraser University, CANADA

## Abstract

Mother-to-child transmission (MTCT) of HIV remains a challenge in Eastern and Southern Africa. Oral pre-exposure prophylaxis (PrEP) is a powerful tool to reduce MTCT, but women face barriers to effective use including those related to inaccurate comprehension of PrEP To understand women’s misconceptions about PrEP and their potential impact on PrEP use, we conducted interviews with 33 pregnant and lactating women in Malawi using PrEP, and ten PrEP counselors and clinicians. The results indicate that, although pregnant women generally understood PrEP’s features and functions, many held misconceptions that persisted over the course of their PrEP use and impacted their perceptions and use of the medication. Some women erroneously believed that PrEP could treat and prevent sexually transmitted infections other than HIV, which motivated some to keep taking PrEP while motivating others to stop using PrEP once their STI had resolved. Some were confused about PrEP’s function, with some believing it was the same as antiretroviral therapy for HIV treatment, and others believing that PrEP could be used for overall enhancement of health. Rarer misconceptions included fears that PrEP was connected to satanic practices, could cause cancer, or was solely for individuals engaged in sex work. These misconceptions stemmed from a mix of prior knowledge, societal influences, and miscommunications during counseling sessions. Ensuring accurate knowledge and addressing common misconceptions head-on is crucial to promote continued PrEP use among women. Both clinic- and community-based communication efforts with a particular focus on the difference between PrEP, STI treatments, and ART are needed.

## Introduction

Eastern and Southern Africa account for the highest number of new human immunodeficiency virus (HIV) acquisitions globally and face persistent challenges in preventing mother-to-child transmission [1]. In Malawi, acute maternal HIV acquisition during pregnancy and lactation are responsible for an estimated 45% of mother-to-child HIV transmission [1]. HIV pre-exposure prophylaxis (PrEP) during pregnancy is a critical tool for prevention of vertical transmission of HIV. The World Health Organization and Malawi guidelines recommend offering oral PrEP to individuals at high likelihood of exposure to HIV acquisition, including pregnant and lactating women [2,3]. Oral PrEP with emtricitabine-tenofovir disoproxil fumarate (FTC-TDF), when taken with high adherence, demonstrates high efficacy in preventing HIV acquisition [4,5]. However, maintaining protective drug levels for cisgender women requires them to use PrEP as prescribed by their provider [6,7]. Unfortunately, studies have shown that women, including during pregnancy, often fall short of taking PrEP as recommended [5,8–10].

Accurate understanding of PrEP is important to promote women’s using it as prescribed. Commonly held misconceptions and misinformation about PrEP can significantly reduce women’s use of PrEP [11,12]. A survey among women in the United States revealed that women held inaccurate beliefs about the safety, efficacy, or proper use of PrEP [13–15], including confusion about whether it is a medication designed to prevent HIV or a live vaccine containing HIV, all leading to low uptake of PrEP [15]. In Uganda, misconceptions about PrEP’s effectiveness, mechanisms of action, side effects, and expected outcomes have discouraged its initiation [16]. In clinical trials in other African settings, including Malawi, misconceptions about the dapivirine vaginal ring, including the ring causing infertility and cancer, have led to nonadherence among participants [17–20]. The extant literature indicates the potential impact of misconceptions on PrEP adoption and use, but significant gaps in this literature remain. Studies to date are limited in scope and have not thoroughly investigated the occurrence and impact of misconceptions on PrEP use among pregnant and lactating women in settings with high prevalence of HIV where PrEP use is particularly impactful. Nonetheless, studies on non-HIV prevention methods suggest that myths, misinformation, and misunderstandings can impede the acceptance and utilization of biomedical prevention methods. Misunderstandings influenced by social networks have been shown to decrease uptake and increase discontinuation of contraceptives methods and human papillomavirus (HPV) vaccines [21–28]. In Malawi, misconceptions that contraceptives methods can cause tumors, cancer, or infertility, have deterred women from initiating or continuing contraceptive use [21–23].

With these insights into the challenges posed by misinformation, this qualitative study aims to deepen our understanding of perceptions and specific misconceptions about oral PrEP among pregnant and lactating women in Malawi, and to assess how these misconceptions influence their use of PrEP. By exploring these factors, we seek to inform improved PrEP education and counseling efforts for this important population.

## Methods

### Ethics statement

Study procedures were approved by the Malawi National Health Science Research Committee and the University of North Carolina at Chapel Hill Institutional Review Board. The pilot trial was registered on www.clinicaltrials.gov (NCT04330989). All participants provided written informed consent prior to study procedures. For illiterate participants, a literate impartial witness was present during the consent process.

### Study context

We present an analysis of data collected in the context of an ancillary study with women participating in the Tonse Pamodzi 2 PrEP pilot trial. The trial enrolled pregnant women in Lilongwe, Malawi, who had indications of a higher likelihood of exposure to HIV and were interested in starting daily oral PrEP. Pregnant women 18 years or older without HIV but with indications of high likelihood of exposure were recruited from Bwaila District Hospital based on specific exposure criteria, such as having a partner living with HIV or unknown status, multiple sex partners, sexually transmitted infection (STI) diagnosis, postexposure prophylaxis (PEP) use, shared injection material or equipment use, or unspecified HIV exposure concerns. Detailed eligibility criteria are published elsewhere [29,30]. All participants received counseling regarding their HIV exposure concerns and how PrEP could mitigate them. They were educated about the functions of daily oral PrEP, the importance of adherence, potential side effects, and safety measures. PrEP prescriptions were provided during the enrollment visit, along with additional information about dosage, efficacy, duration of use, and adherence strategies. Participants were randomly assigned to receive either standard support for PrEP or a combination adherence strategy that included Integrated Next Step Counseling and an optional orientation for a participant-selected adherence supporter. All women underwent comprehensive counseling regarding their individual HIV exposure concerns and the potential exposure reduction associated with PrEP usage. Further details about the content of the pilot trial intervention can be found elsewhere [29,30]. As part of the informed consent process, they were provided with detailed explanations about the mechanism of action of PrEP, the critical significance of consistent adherence, potential side effects, and the safety profile of the medication. Subsequently, upon enrollment, participants were issued prescriptions for PrEP and received additional educational sessions on PrEP functionality, dosing guidelines, effectiveness, duration of use, and various strategies to ensure proper adherence.

### Recruitment and data collection

Women already enrolled in the pilot trial were recruited to participate in this ancillary qualitative study between 5 November 2020 through 18 June 2021. We recruited 30 women to take part in individual in-depth interviews (IDIs) to understand how they decided to use PrEP and their experience using it. We purposively recruited women from the pilot trial for this sub-study to ensure variation in their partners’ HIV status, self-reported adherence level, and intervention arm. We also recruited women (n = 3) who declined to use PrEP to understand the barriers to PrEP initiation. In addition, PrEP counselors and clinicians involved in the pilot trial (n = 10) were recruited by invitation to explore their experiences interacting with the trial participants before and during PrEP use. Counselors and clinicians were eligible for participation if they were at least 18 years of age and had counselled at least one pregnant woman about PrEP use in the pilot trial at the time of recruitment.

Women completed the IDI an average of 102 days after enrollment in the pilot trial (range: 59–239 days) and interviews lasted 25–40 minutes. All IDIs were conducted in Chichewa by a trained female qualitative research assistant fluent in Chichewa and English using a semi-structured interview guide. Interviewers completed training on the study objectives, research questions, interview guides, and conducted practice interviews with the guides prior to data collection. The interview guide for pilot participants included questions pertaining to women’s prior knowledge of PrEP, current understanding of PrEP’s functionality, and their motivations for PrEP use. Interview guides for counselors and clinician questions included questions about: their perceptions of women’s concerns about PrEP and how these concerns influenced PrEP use, support that they provided to women taking PrEP, and barriers that women faced to PrEP use. All IDIs were audio recorded, transcribed, and translated to English.

### Data analysis

In our thematic analysis [31], we initially developed a deductive codebook to capture the *a priori* interview topics from questions in the guide related to women’s decision-making about PrEP use. As we engaged in the coding process, we identified recurrent misconceptions regarding PrEP’s use and function. This realization prompted us to inductively revise our codebook to include these emerging themes and conduct a second round of coding. This phase of the analysis specifically focused on misconceptions about PrEP held by women and how these misconceptions influenced women’s perceptions of and use of PrEP in the pilot study. We defined “PrEP misconceptions” as “any scientifically inaccurate understanding of PrEP’s function, use, or features”. In addition to exploring how misconceptions affected women’s initial and ongoing PrEP use and experiences, we examined women’s concerns about and their broader understanding of PrEP. We also looked at codes of women’s feelings toward PrEP use, motivators and barriers to both initiating and continuing PrEP, the sufficiency of information and support provided. To systematically examine these themes, we created matrices to summarize findings by topic. Higher level matrices were used to summarize findings across participants and to identify relationships between findings. All transcripts were coded using NVivo version 12 software. The interviewers and study coordinator double-coded 20% of transcripts and reconciled discrepancies prior to further coding. One investigator read all interview summaries, field notes, and transcripts for the subset of participants. The analysis procedures and analytic memos were discussed with another investigator throughout the process.

## Results

A total of 33 pregnant women took part in the study. The median age was 25 with a range of 18 and 40 (Table 1). Among these women, 14 reported having a partner living without HIV, while 6 reported being in sero-different relationships. Twenty-seven of the women had been eligible for PrEP due to having an STI diagnosis and 4 for having multiple partners. Of the women who used PrEP, 15 of them had some primary level education and 6 had completed secondary school. Lastly, 10 PrEP counselors and clinicians were interviewed.

**Table 1:** Sample characteristics (n = 43).

	N
**Pregnant & lactating women**	
Median age (range)	25 (18-40)
Using PrEP	30
Declined PrEP	3
Reported partner HIV status of PrEP users (n = 30)	
*Living with HIV*	6
*Living without*	14
*Unknown*	10
PrEP eligibility reasons (past 12 months, not mutually exclusive)	
*STI diagnosis*	27
*Partner of unknown HIV status*	8
*Partner living with HIV*	7
*Multiple sexual partners*	4
Highest Level of Education (n = 30)	
*Some Primary*	15
*Completed primary school*	3
*Some secondary school*	6
*Completed secondary school*	6
**PrEP counselors and clinicians **(n = 10)	
Participant type	
*PrEP counsellor*	3
*Nurse*	5
*Physician*	2

In the interviews, participants demonstrated good comprehension of PrEP; however, some women held misconceptions which they discussed having an impact on their perceptions and use of PrEP. Clinic staff, including counselors, nurses, and clinicians, observed that some women struggled to comprehend the information, leading to these misconceptions about PrEP and its capabilities. Below, we describe the primary PrEP misconceptions held among women, their origins, their perceived effect on PrEP use, and recommendations from PrEP counselors and clinicians to mitigate these misconceptions in the future.

## Types of misconceptions

### STI confusion

Some women expressed a belief that the role of PrEP went beyond HIV prevention, mistakenly believing that PrEP could treat a wide range of STIs. For example, the participant below indicated that she was interested in initiating PrEP because she believed that her STI would be treated by PrEP.

*“I accepted [to use PrEP] because I wanted the disease [STI] to disappear and not reappear.” [82039]*

Another woman reported a positive experience with taking PrEP, noting that she felt reassured about her overall sexual health despite her previously diagnosed STI:

*“It [taking PrEP] is going well because I no longer... I no longer have genital ulcers so that is going on well for me.” [82097]*

The belief that PrEP treats STIs was not limited to the women themselves, but sometimes extended to the safety of their unborn children. Some women, such as the participant quoted below, believed that taking PrEP would prevent her unborn child from contracting any STIs she might contract during the pregnancy:

*“It [PrEP] was to protect the baby from the Candida since, the baby was already in my womb” [82118]*

PrEP function was sometimes extended to not only treating STIs that were already diagnosed but also preventing future recurrence of the same or new STIs. Indeed, clinic staff agreed this misconception was widely spread among women, stating that women believed the medication was given for treatment or prevention of STIs, not HIV prevention:

*“So many women, to them when they were taking PrEP, they thought that they are being protected from STIs. They did not really understand that PrEP is protecting them from HIV. Because a good number of them could say, ‘We have been given medication to protect us from STIs.’” [Counsellor, 002]*

### Confusion with HIV treatment

PrEP was also sometimes misperceived as an HIV treatment, often mistaken with antiretroviral therapy (ART). For some, this was related to confusion about why PrEP was needed if they were not diagnosed with HIV. While this belief went away with time and counseling in the study, it initially led to some mistrust in healthcare providers, with women believing that perhaps the healthcare providers were lying to them about their HIV status:

*“I had concerns that maybe they are not telling me the truth I have HIV and they simply don’t want to tell me. Maybe these are antiretrovirals (ARVs), and they are worried that I am going to be very worried maybe they are hiding something, why are they giving me this medication, so if I have HIV why now, maybe I had it all along.” [82053]*

Study staff also observed participants beliefs that they must be living with HIV if they are taking ARVs as PrEP. Despite explanations from counselors, some individuals still associated ARV medications with HIV treatment, leading to confusion and questioning of PrEP use.

*“Usually because they are human, so even if you explain they could not understand. They were just thinking that we had found them with HIV and that they were given ARVs simply because we were saying it’s an ARV regimen. So, when they heard the word ARV, they thought that we were giving them ART. So, they would ask ‘You said I am HIV negative then why are you giving me PrEP?’” [0001, Nurse]*

### Improvement in overall health

Some women believed that the use of PrEP led to improvements in other aspects of their health and wellbeing, including changes in their appearance and an increase in appetite and energy. They often stated that their health was “much better” and that they fell sick less frequently since starting PrEP:

*“I used to often get sick but ever since I started taking this medication it has all stopped… I would at times feel like I had malaria and cloudy eyes. I would go get tested for HIV but was always negative. Ever since I started taking this medication my health is much better.” [82114]*

In reference to her health improvement, one woman explained that before taking PrEP, she *“used to be lazy and weak,”* but is now *“strong and in good health”* due to her PrEP use [82123].

### Other misconceptions

While the above-mentioned misconceptions were related to positive perceptions of PrEP, some negative misconceptions about PrEP were discussed among women. Though not common, some participants associated PrEP with satanic practices due to the blood draws in the study, while others believed that PrEP was used to intentionally infect women with HIV under the pretense of prevention.

*“When I heard it for the first time from other people, they said the medicine would infect you with HIV.” [82122]*

*“At first it was hard because I was like maybe they would do something scary to me... I thought that they would take me to some satanic place, so I was really concerned… I thought they would take so much blood and take me to some place, I was really scared... So how did you know that these are not satanic worshipers?” [82114]*

Other participants believed that perhaps PrEP was meant exclusively for women involved in sex work or as a precaution for those who had experienced a recent sexual assault, often confusing it with PEP.

*“I heard that if…my young sister was raped and was given this drug [confusing it with PEP]. That was my first time to hear about it. I used to hear also that when healthcare workers are working and get hurt while helping someone are given PrEP [meaning PEP] to protect them.” [82023]*

Moreover, one participant initially believed the use of PrEP would cause cancer or damage to internal organs due to the accumulation of the medication in the body, especially because PrEP was not treating an infection.

*“On taking medicine when you do not have the virus…That they [PrEP] could damage you inside… Because of the large quantity of this medicine in the body. I thought maybe they could cause other diseases inside me…Cancer.” [82020]*

Several participants were also concerned about the use of PrEP during pregnancy and thought using the medication would lead to miscarriages, or birth defects.

*“I thought that I would miscarry or lose the baby or deliver a preterm baby.” [82013]*

Lastly, though it did not come up among the women themselves, a clinician talked about some women who believed PrEP could potentially make them sterile due to the medication being given specifically to women and not men.

*“Stories that the medication [PrEP] just wants to make the women sterile so that they are not able to have children like a way of dealing with reproduction, they were saying why are they giving this mostly to the women and not the men.” [Nurse, 0010]*

## Origins of misconceptions

Some of the misconceptions above, particularly the negative ones (e.g., PrEP being associated with satanic practices), were often rooted in information participants had previously received about PrEP. Participants who joined the study with some prior awareness of PrEP (and sometimes, prior misconceptions) had gathered their PrEP information from conversations with friends, family, radio, or community members, highlighting the influence of the community’s pre-existing beliefs on HIV treatment and prevention.

*“When I heard it for the first time from other people, they said the medicine would infect you with HIV… Before I heard here about PrEP I heard women [in the community] say that this PrEP is a satanic thing.” [82122]*

PrEP-naïve women (those encountering PrEP information for the first time during the study) held a spectrum of misunderstandings. Study staff believed that the limited education and familiarity with the healthcare system coupled with the lack of PrEP awareness in the community further worsened these misconceptions. Staff shared that for the majority of women the study was the first they had heard of PrEP, thus leading women to believe rumors about PrEP heard in the community. Other misconceptions were often indicative of participants misunderstanding the PrEP counseling provided in the study. In fact, some participants often attributed their misunderstandings directly to the information they received from study PrEP counselors and clinicians:

*“They [counselors] said that we need to be taking PrEP to be protected from HIV infection and also to protect the unborn baby from HIV infection and candida.” [82118]*

Some women also described lack of information as having contributed to their misunderstandings. While they had questions or concerns during counseling, they hesitated to ask questions because they felt overwhelmed, leading to misunderstandings of the information provided.

*“I had questions about what they wanted the drugs to do with our bodies. I had never heard about the drugs, and I didn’t know.”* [82039]

Finally, the fact that many women had an STI diagnosis led some women to believe that PrEP was meant to treat or prevent STIs. Clinic staff attributed this misconception to the fact women were recruited to join the study because of their STI diagnosis and started STI treatment around the same time as enrolment. As such, some women had the perception that PrEP was treating STIs rather than the actual STI treatment they received:

*“Half of them understood about PrEP but others were confused because they were high risk with STI’s, and they thought PrEP was part of the STI treatment that was one thing even though you explain to them you could find that they are still saying the same on the next visit. They had a combined element that the drugs will prevent HIV infection and treat STI’s.” [Nurse, 006]*

## Impact of misconceptions on PrEP Use

For some, misconceptions had an important impact on their PrEP use experience. The belief that PrEP would treat STIs or that it improved their overall wellbeing motivated them to adhere to their PrEP regimen. They feared that missteps in not taking the pills daily could lead to a recurrence of STIs or them reverting to their previous health status.

“*You need to remember to take it [PrEP] daily, at the same time, or else the infection [STI] can come back.” [82019]*

Interestingly, some misconceptions leading to positive PrEP perceptions, including the idea that PrEP had treated diagnosed STIs or improved their overall wellbeing, persisted even when counselors or clinicians provided accurate information. Some women experienced a sense of cognitive dissonance when discussing improvements in their STIs during the interviews, attributing these changes to PrEP use. Despite PrEP counselors and study staff clarifying that PrEP does not protect against other STIs beyond HIV, these women believed their symptom improvements or lack of reinfection must have resulted from their PrEP use, as described by this participant:

*“I know you say that PrEP doesn’t protect against other STIs, but I have not had any other infection [since taking PrEP].” [82106]*

Clinic staff emphasized the importance of routine comprehension checks during counseling sessions to identify gaps in understanding. In their encounters with the women in the study, counselors and clinicians spoke of encouraging women to ask questions and seek clarification but acknowledged that, at times, the complexity of PrEP information overwhelmed participants. Some counselors discussed attempts to educate women who were still confused about why they needed PrEP if they were not living with HIV.

*“Some of them did not understand why they were getting medication as if they had HIV…So, we were clarifying that we are not putting you on ART. So, it was that at home people would say that taking the medication meant that they had HIV and that were at the clinic were hiding it from them. So, we were clarifying in those.” [Clinician, 009]*

Additionally, some study staff spoke on the importance of providing additional counseling to women who were motivated to use PrEP because they thought it would cure STIs and wanted to stop when their STIs seemed to be cured, having to reexplain the functionality of PrEP.

In most cases, misconceptions more commonly led to positive perceptions of PrEP than not. Study staff observed that women, particularly those with lower levels of education, often struggled to grasp the information provided and consequently delayed initiating PrEP due to uncertainties in their understanding. This was particularly pronounced among women who confused PrEP with HIV treatment, likely due to limited community knowledge of PrEP and its similarity to ART. This led women to fear that they would be stigmatized as living with HIV by their social networks. While most women approached for recruitment agreed to use PrEP, not all felt comfortable disclosing this decision to their social networks. Study staff also noted instances in which participants were puzzled by the need to take PrEP if they were living without HIV, requiring additional motivation to maintain daily adherence.

*“Some women complained that is was hard for them to be able to be taking PrEP every day they even mentioned that their interest declines because you can see that you are just taking the medication and you are not sick so it was still hard for them to be taking the medication every day while they were not sick. Some even had challenges with adherence after we had discussions.’ [00007, Nurse]*

For two participants recruited for interviews as PrEP decliners, it was their misconceptions that led them to decline PrEP. The first participant declined due to confusion about the necessity of PrEP when her STIs were treated. She believed that PrEP was only for pregnant women diagnosed with STIs. Thus, she believed she did not need it since her STIs were treated, and she feared relationship conflicts stemming from accusations by her partner that she was living with HIV:

*“This really scared me that I should be taking medication for 6 months since they [clinicians] have tested me for HIV and other tests have all come out negative, why would they want me to be taking these medications… So, when I explained to my husband when I got home, he told me that I should not be taking this medication… [He said] ‘they tested you for HIV and vaginal discharge which are all negative so why do they want to give you medication, wouldn’t this medication bring you other problem?’ He said these medications look like ARVs and they are taken by a person who is HIV positive so ‘maybe you have HIV, and you are just lying to me’.” [82099, Decliner]*

The second participant declined because of confusion about the need for PrEP when she was not living with HIV and concerns about stigma from her family as illustrated in the quote below:

*“I was concerned to say what is this for because I have not tested positive and now you say I should start taking the medication… I was like they have not found me with HIV... Now they want me to be taking the medication, what is this medication going to be helping with? It was that it’s obvious I might have the virus, so when people see that I am taking the medication they would say I have the virus even though I tested negative.” [00001, Decliner]*

Misconceptions or concerns about the effect of PrEP use on the health of the pregnant women and their unborn child diminished over time, especially when pregnancies progressed without challenges and healthy babies were born. Study staff provided suggestions to mitigate misconceptions in the future. Many staff felt that about half of the women did not fully understand the information shared and thus, emphasized the importance of community awareness and advocated for disseminating PrEP information beyond healthcare facilities – reaching out to the broader community to ensure that accurate knowledge is accessible to all.

*“Information on PrEP is mostly discussed in the health facilities where the women patronize but I think that this information should go out to everyone because of what I had said because for people to make decisions they rely on help from other people. It would be helpful if these other people also had some sort of information whether from the radios or in whatever way it would mean that they have some sort of knowledge so that when they are giving advice it is well informed.” [Counselor, 004]*

## Discussion

Our findings revealed that some women who used PrEP during pregnancy and lactation in Malawi harbored misconceptions about the purpose of PrEP, despite receiving counseling prior to and during use. These misconceptions included views that PrEP was a treatment for STIs, could protect unborn children against STIs, and was a treatment for HIV and unrelated health issues. A few women were also confused about their HIV status, often believing they were found to be living with HIV, based on the belief that they were being given PrEP as an HIV treatment. While most of these misconceptions originated from women’s own misunderstandings of the information at the time of PrEP initiation, other misconceptions originated from prior information they had learned about PrEP from sources in the community. The impact of these misconceptions on women’s PrEP use experience was diverse with some beliefs leading women to feel motivated to use PrEP, and other misconceptions leading women to hold concerns that prevented their initiation or continued use of PrEP. To address misconceptions, counselors emphasized the importance of routine comprehension checks during counseling sessions and community-wide education to ensure accurate PrEP knowledge reaches all, supporting informed decision-making in PrEP use.

In light of emerging biomedical HIV prevention methods such as the dapivirine vaginal ring and long-acting cabotegravir injectable PrEP, it is essential to understand the barriers women face when considering these options. Safety data indicate that both methods are safe to use during pregnancy and lactation [32–36]. Injectable lenacapavir is still being studied for safety in pregnancy and lactation, but may become available to this population in the future [37]. This expands the landscape of PrEP options available to women, allowing for more personalized decision-making that considers individual health needs and preferences. Further, as the landscape of options becomes more complex, there is even more new information that women must learn, increasing potential for misconception and increasing the need for community education. Addressing misconceptions surrounding newer options can further empower women, fostering an environment where they feel confident to make informed decisions regarding their sexual health [38]. Thus, alongside improving awareness about oral PrEP, it is crucial to engage in community education about these new methods, ensuring that women can weigh all available options in their decision-making process regarding HIV prevention [39].

Indeed, integrating community-wide education efforts (including tailored marketing strategies) and conducting routine comprehension checks during counseling sessions has been proven not only to promote accurate PrEP knowledge but also to empower individuals to effectively navigate social pressures and challenges related to PrEP use [16,40,41]. In addition, some participants in our study attributed various outcomes, such as improved STI symptoms, better general health, or the prevention of STIs to PrEP use, despite being educated that it only protects against HIV. Counselors in our study thought that the misconception of PrEP treating STIs could have emerged due to women being eligible to join the study as a result of their STI diagnosis. In implementation settings, healthcare providers must work to ensure that women know the differences between and functions of STI medications and PrEP. Similar findings in another study revealed misconceptions among PrEP users regarding its effectiveness against other viral infections beyond HIV, and thus led participants to feel PrEP was unnecessary since they had a strong innate immune system [16].

As suggested by the study counselors, implementing routine comprehension checks that take into account patients’ literacy levels could assist in identifying and rectifying misconceptions. In a US study with patients living with HIV with low literacy levels, patients appreciated healthcare providers who simplified explanations, leading to more accurate knowledge and trust between the patient and provider [42]. Another study conducted in Uganda found that individuals who declined PrEP attributed their lack of awareness and misunderstandings about the function of PrEP to inadequate explanations by healthcare providers [43]. Adopting an individual-centered counseling approach could help address these misconceptions, and this effort could be facilitated by drawing upon evidence-based practices from other fields. Two systematic reviews offer evidence for effective interventions to increase accurate health knowledge and patient-centered decision-making. One review on contraceptives found that patient decision aids, videos, and written materials could enhance understanding and informed decision-making [44]. A second review of epilepsy interventions found that diverse activities, such as lectures, pamphlets, and media engagement, could contribute to a reduction in stigma and improved knowledge surrounding the condition [45,46].

We also found that community rumors and HIV-related stigma had a profound impact on misconceptions. Similar to other studies, some women in our study initially confused PrEP with ART due to PrEP’s association with ARVs and HIV [16,47,48]. These misconceptions highlight the enduring stigma and misunderstandings surrounding HIV within specific communities, and also led some participants to fear that they would be perceived by their social networks as living with HIV if seen taking PrEP. These findings are consistent with those of other studies showing that anticipated stigma frequently prevents individuals from disclosing their PrEP use to their social networks, depriving them of potential support for optimal adherence [41,43,49–52]. In a study conducted in Uganda, one community’s widespread belief that HIV is untreatable and lacks preventive medicine contributed to low PrEP adherence and doubts about PrEP’s effectiveness [40,53]. Additionally, the use of ARV clinics to introduce oral PrEP has contributed to the stigmatization of its use in some settings, leading to misconceptions that PrEP is a treatment for HIV rather than a preventive measure [49,54,55]. In the Malawian context, it is vital to recognize that such anticipated stigmatization can hinder women’s willingness to initiate and continue to use PrEP [56]. Addressing these misconceptions within healthcare settings is crucial, ensuring that women understand the distinct purposes of PrEP and ART, prior to initiation of use [54]. Anticipated stigma associated with PrEP use, such as being labeled as promiscuous or a sex worker, and the fear of domestic violence if PrEP is confused with ART, are all reported factors that prevent individuals from initiating PrEP use [50,57]. However, other studies have shown that accurate information and positive experiences with PrEP shared by peers in the community can motivate individuals to initiate PrEP [16]. Moreover, other studies have reported the confusion among communities regarding PrEP initiation when individuals are not sick [58].

Some participants reported community beliefs associating PrEP with satanic practices or fear of severe side effects causing miscarriages in pregnant women. Widespread misinformation about the safety of PrEP, including PrEP use potentially causing cancer, miscarriages, or infertility were reported to contribute to short-and-long-term PrEP interruptions among PrEP users in Kenya [59]. A few participants also spoke of the misconceptions of PrEP being only for women who sell sex, aligning with other previous studies [51].

To address these misconceptions, our counselor participants recommended community education campaigns to promote PrEP awareness and accurate knowledge. Recognizing that different levels of social interaction—such as involvement from partners, peers, and community members—can significantly influence women’s decisions regarding PrEP is essential [51,60,61]. Facilitating peer-to-peer counseling and information sharing can enhance understanding and create environments that are not stigmatizing within the community [49,62]. This approach not only fosters trust and open dialogue about PrEP but also empowers women to make informed health decisions [51]. Additionally, engaging communities, involving community leaders and organizations, and promoting access to accurate information through various channels, including social media platforms, can effectively address misconceptions, debunk myths, remove users’ feelings of shame, and create an environment of acceptance and support for PrEP users (41,54,55). This is particularly crucial as new PrEP options, such as the dapivirine vaginal ring and injectable PrEP, are being introduced.

Our findings highlight the range of different misconceptions women have about PrEP and the impact these beliefs can have on women’s use of oral PrEP. These results underscore the need to address these misconceptions to ensure a positive PrEP experience and limit challenges in PrEP initiation and use. Our study results should be interpreted with key limitations in mind. First participant responses in the interviews are subject to potential social desirability bias. For example, women could have felt obligated to say they understood PrEP’s functionality to limit judgement from study staff. To mitigate this bias, participants were reminded that there were no right or wrong answers and that their responses would not affect their participation in the pilot study or their relationship with the study clinic. Second, it is important to note that many women in this study had been identified as PrEP candidates based on having had a recent STI diagnosis and thus may be more likely to confuse PrEP and STI treatments than women identified as PrEP candidates for other reasons. However, STI diagnoses are likely to trigger PrEP referral in practice as well, thus we would anticipate STI treatment and prevention-related misconceptions to be common in clinical populations. Lastly, our participants were recruited from urban and peri-urban areas, meaning that the results may not be generalizable to women in rural settings, as perspectives and experiences may differ among women and even PrEP counselors in rural and urban areas.

## Conclusions

Our study highlights the significant role of misconceptions in women’s perceptions and use of oral PrEP and emphasizes the importance of addressing these misunderstandings to ensure PrEP users, especially during the perinatal period, have accurate knowledge about PrEP functionality when deciding whether and how to use PrEP. To overcome misconceptions, it is necessary to adopt an individual-centered approach during counseling, provide comprehensive information, and ensure patients feel empowered to ask questions and voice their concerns. Community education and awareness programs, involving community leaders, organizations, peers, and healthcare providers, can also play a crucial role in dispelling misconceptions, combating stigma, and disseminating accurate information about PrEP. As new PrEP options emerge, it will be vital to engage local clinics and communities to foster accurate community knowledge and support for PrEP users.

## Supporting information

S1 Text Inclusivity in global research questionnaire. (DOCX)
